# Arachidonic Acid Metabolism and HETEs-PGs Imbalance in *L. infantum* Infection: Implications for Visceral Leishmaniasis Progression

**DOI:** 10.1021/acsomega.5c04179

**Published:** 2025-09-12

**Authors:** Yasmin Monara Ferreira de Sousa Andrade, Astrid Madeleine Calero Goicochea, Flávio Henrique Jesus-Santos, Jonathan Luís Magalhães Fontes, Bianca Ramos Mesquita, Caroline Vilas Boas deMelo, Nicole Hlavac, Icaro Bonyek-Silva, Manuela da Silva Solcà, Deborah Bittencourt Mothé Fraga, Adriana Ferreira Lopes Vilela, Carlos Arterio Sorgi, Washington Luis Conrado dos Santos, Théo Araújo-Santos, Valeria M. Borges

**Affiliations:** † Federal University of Western Bahia, Barreiras 47808-021, Brazil; ‡ Gonçalo Moniz Institute, Oswaldo Cruz Foundation, Salvador 40296-710, Brazil; § Federal University of Bahia, Salvador 40170-110, Brazil; ∥ Federal University of Rio Grande do Sul, Porto Alegre 90040-060, Brazil; ⊥ University of São Paulo, Ribeirão Preto 14040-903, Brazil

## Abstract

Visceral leishmaniasis (VL) alters lipid metabolism, impacting the production of bioactive compounds like eicosanoids, which regulate inflammation—a key aspect of the disease. This study investigated eicosanoid production in Golden Syrian hamsters infected with *Leishmania infantum* for five months. The infected animals developed splenomegaly, increased creatinine, elevated liver transaminases, granulomas, white pulp hypoplasia, and portal infiltrates. Arachidonic acid (AA) mobilization was elevated in the spleen and liver but unchanged in plasma. Liquid chromatography mass spectrometry (LC–MS/MS) analysis revealed increased hydroxyeicosatetraenoic acids (HETEs) in the spleen, while prostaglandin (PG) E_2_, 2-keto-PGE_2_, and PGD_2_ were reduced. Notably, splenomegaly, higher HETEs, and lower PGs levels correlated with parasite load, suggesting *L. infantum* manipulates these mediators to promote inflammation and its persistence. The imbalance between HETEs and PGs seems crucial for VL progression, highlighting the need for further research into the mechanisms driving disease pathogenesis.

## Graphical abstract

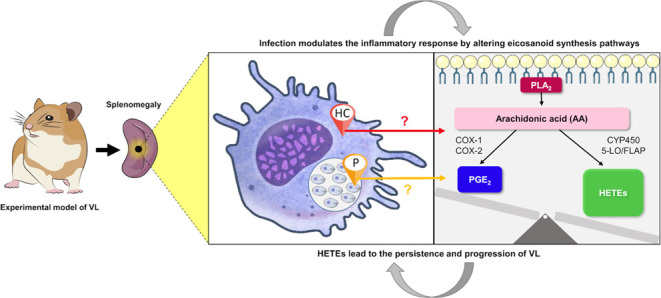

## Introduction

Leishmaniasis is still one of the most neglected diseases in the world, mainly affecting poor and developing countries.^1^ The disease is caused by different species of protozoa of the genus *Leishmania*, which are transmitted to animals and humans through the bite of sandflies.^2^ Among the leishmaniasis, VL, which has *Leishmania infantum* as its etiological agent, is considered the most serious form because it affects organs and hematopoietic tissues and if not treated properly can cause the death of patients.^1,2^

Understanding disease progression is crucial for developing effective treatment options. In this regard, the use of small animal models is essential. The Golden Syrian hamster (*Mesocricetus auratus*) is widely recognized as an experimental model for VL due to its ability to accurately replicate the clinical and pathological features of the disease in humans, characterized by progressive increase in the parasite load in the visceral organs, cachexia, marked splenomegaly and hypergammaglobulinemia.^3,4^

During parasite interaction, host cells activate microbicidal mechanisms and produce molecules such as eicosanoids, which regulate cellular processes under both normal and disease conditions and are derived from the oxidation of AA.^5,6^ The production of eicosanoids is initiated by the action of cytosolic phospholipases (cPLA_2_) on cellular lipids, releasing AA molecules that will be transported into the lipid bodies by membrane proteins present in these organelles, such as perilipins (PLIN-1).^5,6^ Then, AA molecules can be metabolized by different enzymes, such as cyclooxygenases (COX), lipoxygenases (LOX) or cytochrome P450-like proteins (CYP450), leading to the production of PG or thromboxane, leukotriene and HETE, respectively.^7^

The role of eicosanoids in the immune response and pathogenesis of VL has been documented, primarily through the identification of PG and leukotriene mediators in the serum of humans and dogs affected by the disease.^8–10^ Recently, eicosanoids from the HETE class were quantified in the serum of patients with cutaneous leishmaniasis undergoing leishmanicidal treatment, with 11-HETE identified as a biomarker capable of accurately predicting and characterizing therapeutic failure.^11^ Additionally, various types of HETEs mediators were primarily identified in the supernatant of axenic cultures of metacyclic promastigotes of *Leishmania* species.^12^

The scientific literature lacks studies that identify and quantify eicosanoids, including HETEs, in the target organs of VL using LC–MS/MS. Furthermore, the role of these metabolites during the host-parasite interaction remains poorly understood. This study, therefore, represents the first report of in situ eicosanoid synthesis and its influence on VL pathogenicity, using Golden Syrian hamsters as an experimental model.

## Results

### *L. infantum* Induced Splenomegaly Without Hepatomegaly or Skin Changes in Hamsters

Splenomegaly was observed in hamsters infected with *L. infantum*, while hepatomegaly was not detected (Figure 1B–D). Additionally, these animals exhibited no alterations in the skin or mucous membranes compared to the control group.

**Figure 1:**
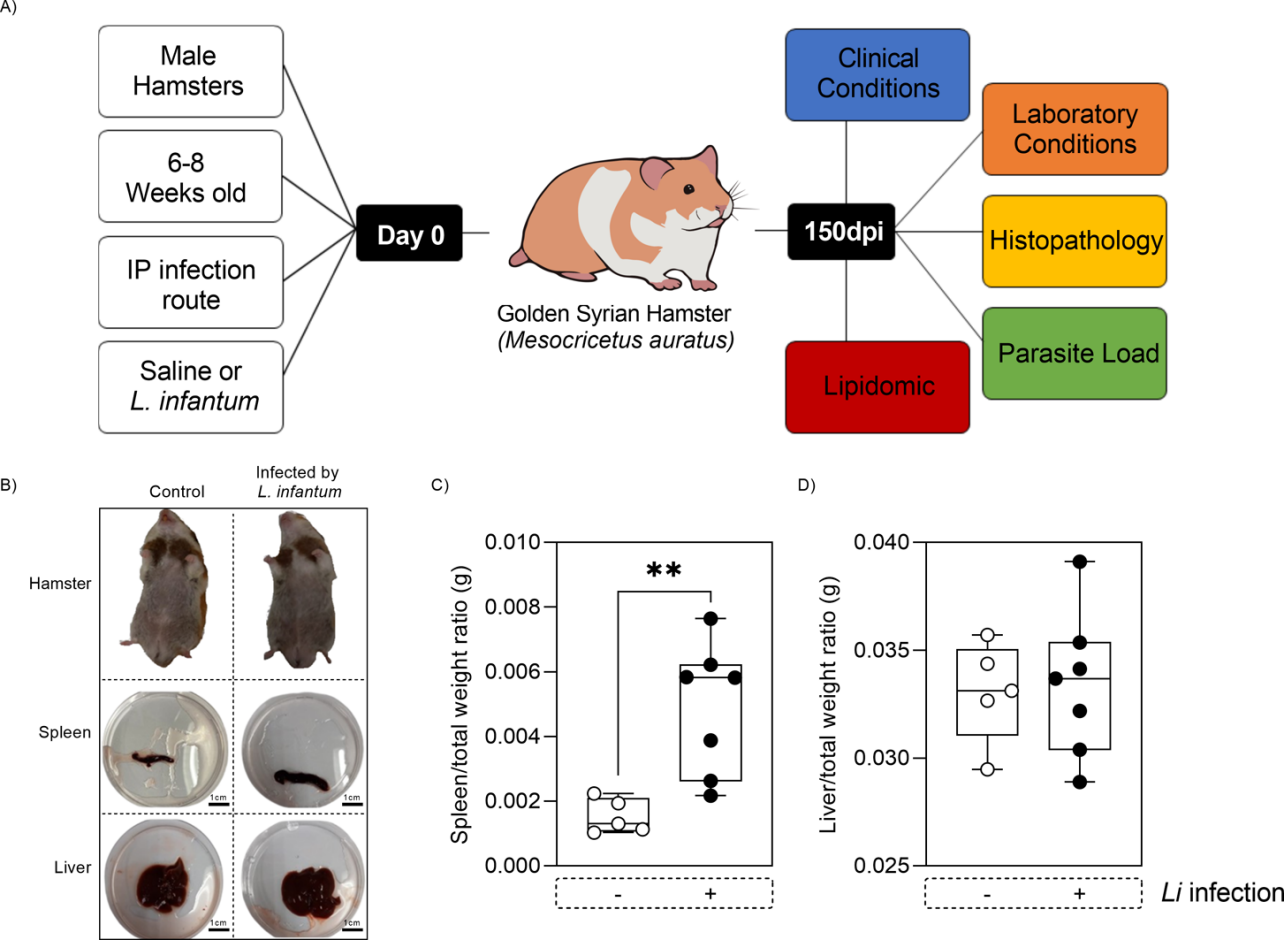
Clinical assessment of Golden Syrian hamsters infected with *L. infantum*for 150 days. (A) Experimental design illustrating the infection of Golden Syrian hamsters with *L. infantum*, and the postinfection evaluation criteria (*n* = 7 animals per group) (BIOART-000278). (B) Macroscopic records of animals from distinct groups and their respective target organs of VL, scale bar 1 cm. Graphs showing variations in spleen (C) and liver (D) weights across the groups. Data are presented as the mean from a representative experiment, with statistical significance indicated by **p < 0.01. IP: intraperitoneal; dpi: days post infection. The negative sign (−) represents uninfected animals, while the positive sign (+) represents animals infected by *L. infantum*.

### Biochemical Alterations in Serum of *L. infantum*-Infected Hamsters

Infected hamsters showed significant increases only in creatinine, ALT, and AST levels compared to controls (Table 1).

**Table 1:** Biochemical Evaluation of Serum Golden Syrian Hamsters Control and Experimentally Infected with *L. infantum* ^a^

Parameters	Unit	Normal range	Control *n* = 6	Infected *n* = 6	*P*-value	Posttest result
Total protein	g/dL	4.5–7.5 g/dL	4.75 ± 0.27	4.73 ± 0.25	0.9146	ns
Albumin	g/dL	2.3–4.3 g/dL	3.31 ± 0.20	3.21 ± 0.19	0.4049	ns
Urea	mg/dL	36.2–50.4 mg/dL^*b*^	44.63 ± 8.59	39.13 ± 6.76	0.2463	ns
Creatinine	mg/dL	0.2–1.0 mg/dL	0.34 ± 0.01	0.40 ± 0.04*	0.0104	*
ALT	UI/L	22–128 UI/L	72.16 ± 27.42	158.4 ± 54.67*	0.0111	*
AST	UI/L	20–150 UI/L	81.70 ± 46.42	200.0 ± 54.95**	0.0087	**
Cholesterol	mg/dL	55–181 mg/dL	72.87 ± 11.50	76.27 ± 14.18	0.658	ns
Triglyceride	mg/dL	72–227 mg/dL	94.22 ± 14.96	87.64 ± 18.05	0.5952	ns
HDL	mg/dL	49.75–66.50 mg/dL^*b*^	56.83 ± 8.58	52.50 ± 5.82	0.3304	ns

a As the data follow a Gaussian distribution, values are expressed as the mean ± standard deviation (SD). Comparisons of biochemical parameters between infected and uninfected animals were analyzed using *T*-tests. *P*-values from posthoc tests are represented as follows: *p < 0.05 and **p < 0.01. Superscript *b* indicates that the normal range was determined from the minimum and maximum values of the control group. *n*: number of animals; ns: nonsignificant

### Histopathological Changes in the Spleen and Liver of *L. infantum*-Infected Hamsters

The splenic microenvironment of hamsters, both infected and uninfected with *L. infantum*, was analyzed for granuloma formation and white pulp hypoplasia, which are characteristic features of VL. Hamsters infected with *L. infantum* showed a higher number of granulomas and a reduced white pulp area. The liver of infected animals also exhibited a higher number of granulomas. Additionally, the presence of portal infiltrates was assessed, revealing larger areas of infiltrates in the infected group (Figure 2).

**Figure 2:**
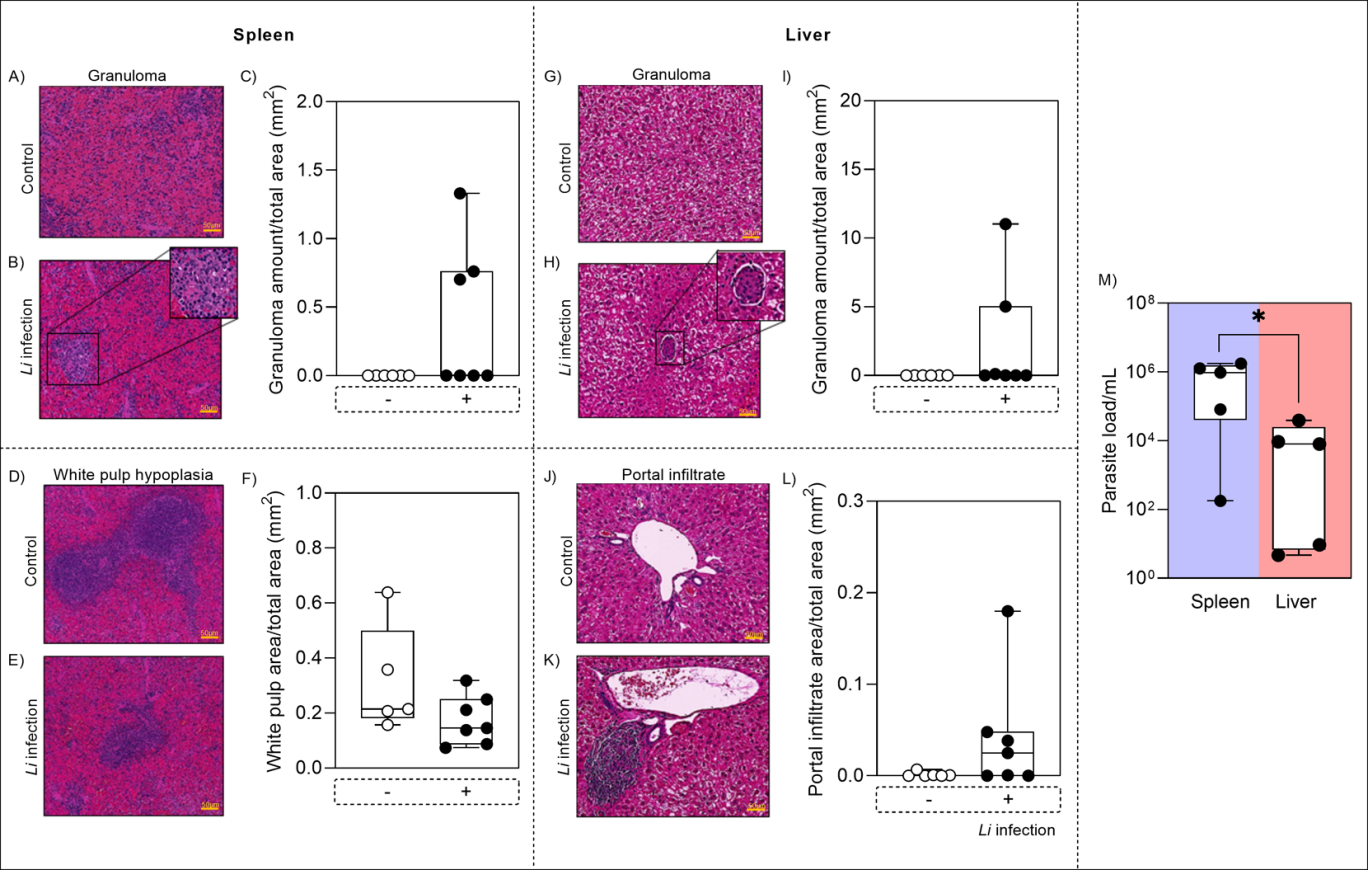
Histological analysis of the spleen and liver in Golden Syrian hamsters infected with *L. infantum*for 150 days. Microscopic alterations include the absence of granulomas in uninfected animals (A) and the presence of granulomas in the splenic parenchyma of infected hamsters (B) (*n* = 7 animals per group). Morphometric analysis of granuloma formation is shown in (C). Another histological feature evaluated in the spleen was white pulp hypoplasia, observed in uninfected animals (D) and in those infected with *L. infantum* WT (E). The corresponding morphometric analysis is presented in graph (F). In the liver, microscopic alterations evaluated included the absence (G) and presence (H) of granulomas in uninfected and infected animals, respectively, with group comparisons shown in (I). Additionally, control animals exhibited no portal infiltrates (J), whereas infected animals displayed areas of portal infiltration (K). The morphometric analysis of this feature is depicted in (L). Finally, the parasite load across VL target organs is compared in (M). Data are expressed as the mean or median from a representative experiment, with statistical significance indicated as *p < 0.05.

### Imbalance between HETEs and PGs in Target Organs of VL

In the target organs of VL, polyunsaturated fatty acid (PUFA) and eicosanoids, including AA, 5-HETE, 8-HETE, 11-HETE, 12-HETE, 15-HETE, LTB_4_, 5-oxo-ETE, 15-oxo-ETE, PGE_2_, 15-keto-PGE_2_, and PGD_2_, were detected (Table S1). Notably, the mediators PGF_2α_ and PGB_2_ were exclusively identified in the spleen. The mobilization of AA was significantly elevated in the spleen and liver of hamsters infected with *L. infantum*, whereas eicosanoid synthesis was predominantly observed in splenic tissue. Among the eicosanoid classes, HETEs were the most abundant in the spleen, with higher levels in infected hamsters. In contrast, the synthesis of eicosanoids such as PGE_2_, 15-keto-PGE_2_, and PGD_2_ was markedly reduced in hamsters with VL. These findings demonstrate that *L. infantum* modulates eicosanoid synthesis in the spleen, leading to an imbalance between HETEs and PGs (Figure 3).

**Figure 3:**
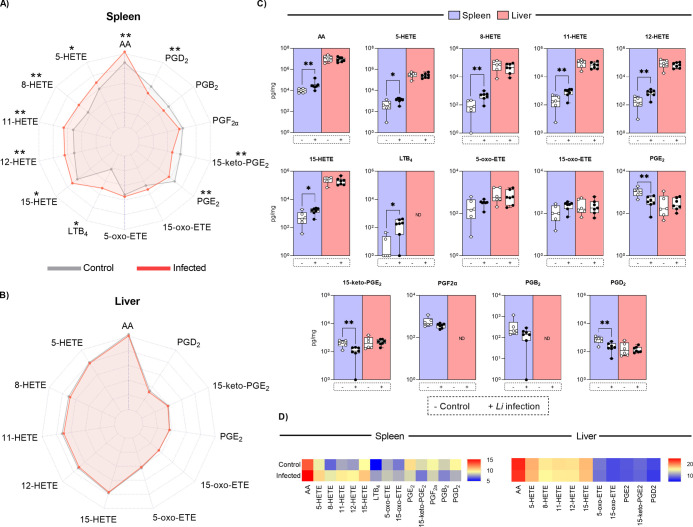
Synthesis of PUFA and eicosanoids in the spleen and liver of hamsters with VL. Spider charts illustrate the production of AA and other eicosanoids in the spleen (A) and liver (B) of the animals (*n* = 7 animals per group). The synthesis of these mediators across distinct groups and target organs can also be observed in the box plots (C) and heatmaps (D). Data are expressed as the mean or median from a representative experiment, with statistical significance indicated as *p < 0.05, **p < 0.01, ***p < 0.001.

### AA Dynamics Link Inflammation, Eicosanoids, and Parasite Load in the Spleen and Liver

In infected spleens, higher parasite load was associated with splenomegaly, elevated creatinine, AST, AA, and HETE levels, and decreased PG levels (Figure 4A). Furthermore, AA is positioned at the center of the interaction between these three factors, suggesting its key role in regulating the inflammatory response and lipid metabolism (Figure 4B). The shift toward HETE synthesis at the expense of PG production highlights an imbalance between these eicosanoids during *L. infantum* infection (Figure 5). Therefore, the modulation of AA and HETEs may play a crucial role in the progression of VL.

**Figure 4:**
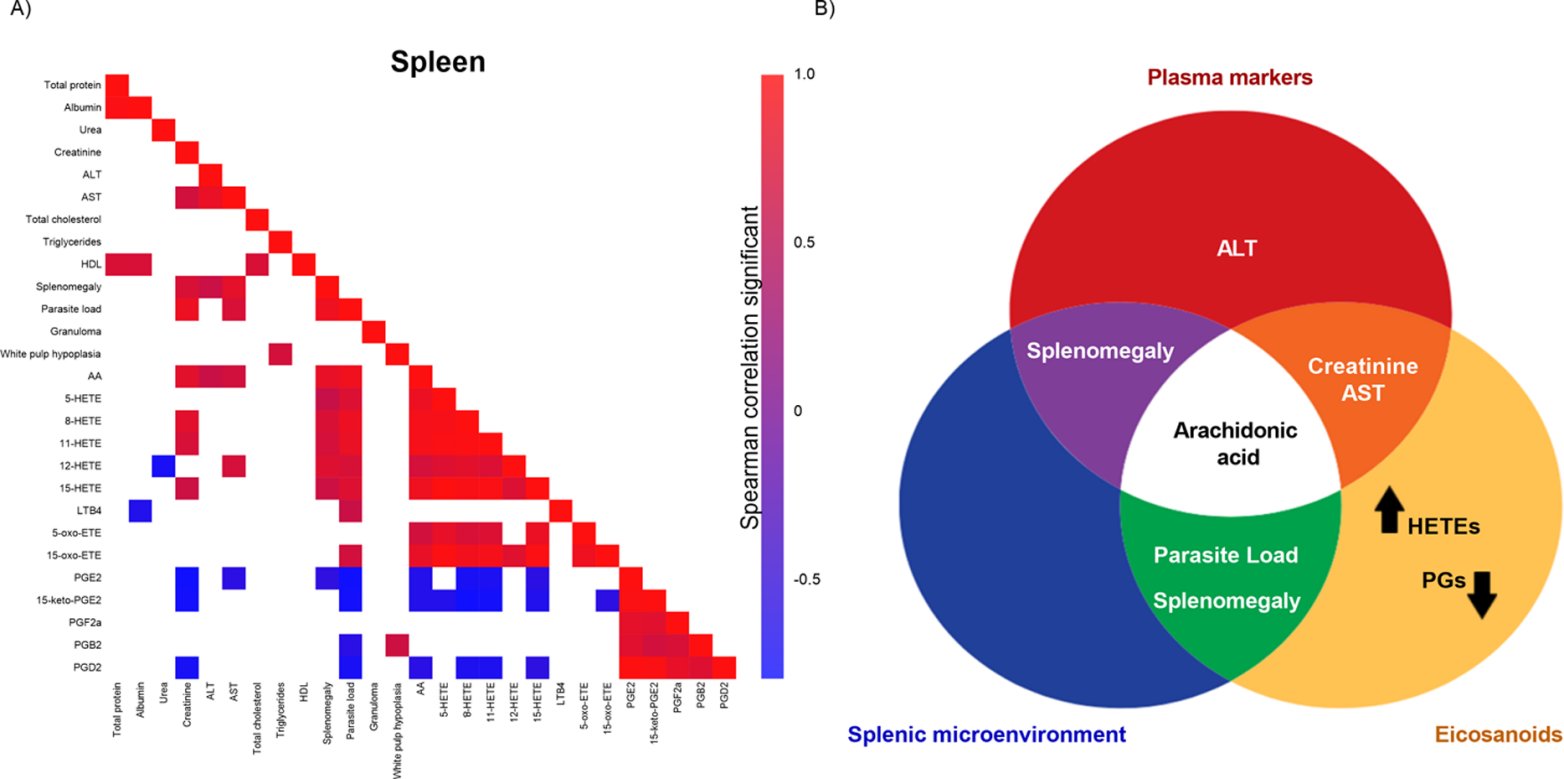
Metabolic and inflammatory changes in the spleen associated with infection and parasite load. (A) Spearman correlation matrix depicting the relationships between various biochemical, inflammatory, and parasite load parameters (*n* = 7 animals per group). Color intensity represents the strength of the correlation, with red indicating significant positive correlations (*p* < 0.05) and blue indicating significant negative correlations (*p* < 0.05). (B) Venn diagram illustrating the overlap between different evaluated categories: plasma markers (red), eicosanoids (orange), and the splenic microenvironment (blue).

**Figure 5:**
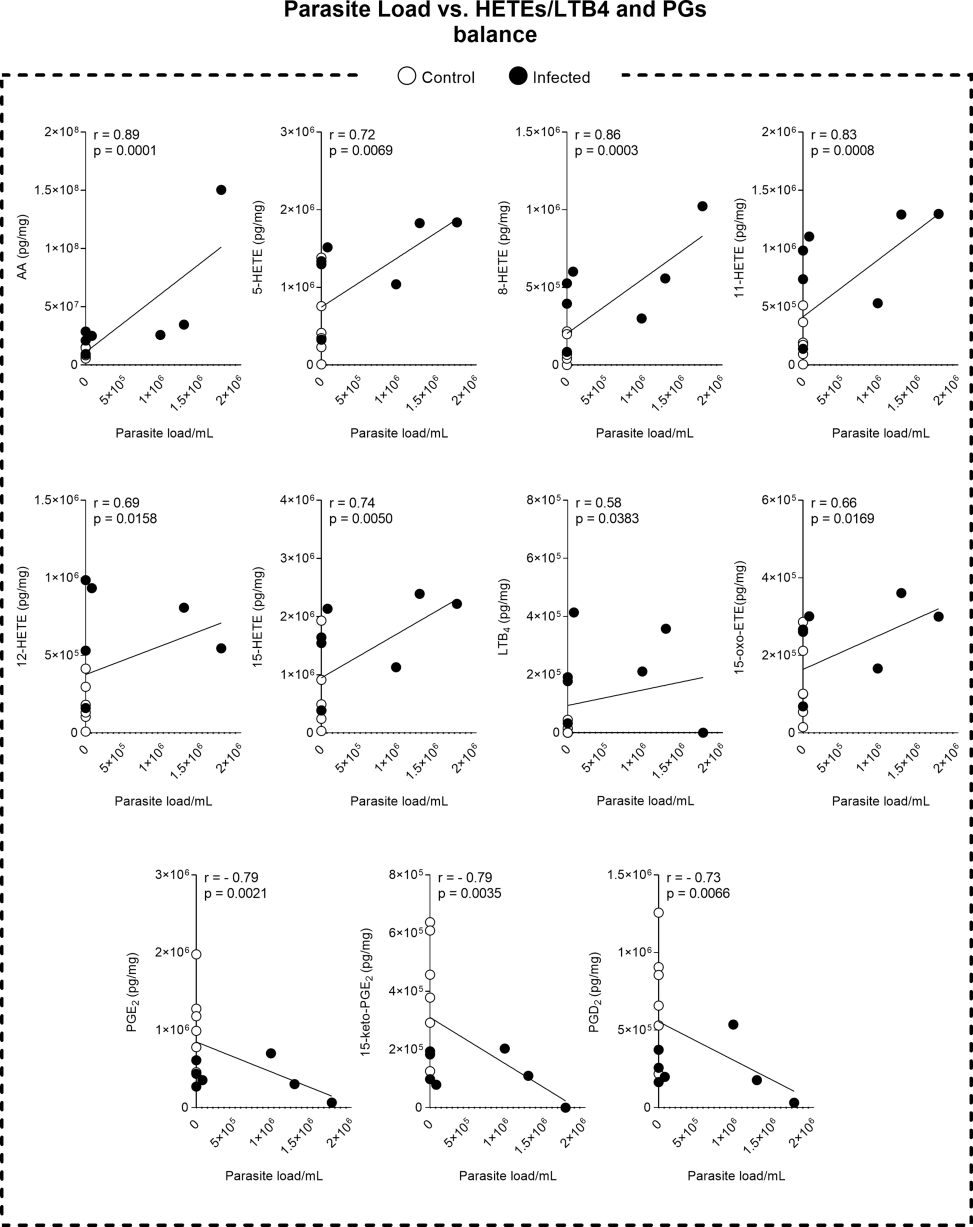
Correlation between parasite load of the spleen and the synthesis of eicosanoids: HETEs, LTB4, and PGs. Correlations were assessed using Spearman’s test. In the graphs, the control group is represented by white circles, while the black circles represent the infected animals (*n* = 7 animals per group). Abbreviations: HETEs: hydroxyeicosatetraenoic acids; LTB4: Leukotriene B4; PGs: prostaglandins; r = Pearson correlation coefficient; *p* = *p* value.

In the liver, the infection resulted in a significant portal infiltrate, which was directly associated with increased plasma ALT levels and reduced in situ HETEs production (Figure 6A,B). This modulation of HETEs may suggest an alteration in the eicosanoid metabolic pathway, likely influenced by the infection, which predominantly localizes in the spleen. Furthermore, a dynamic interplay between the epoxyeicosatrienoic acids (ETEs) and PG eicosanoid classes was observed (Figure 6C), possibly influenced by substrate availability and the need to modulate inflammation. The Figure 7 summarizes and proposes, based on our results, the processes of eicosanoid metabolism involved in the pathogenesis of VL.

**Figure 6:**
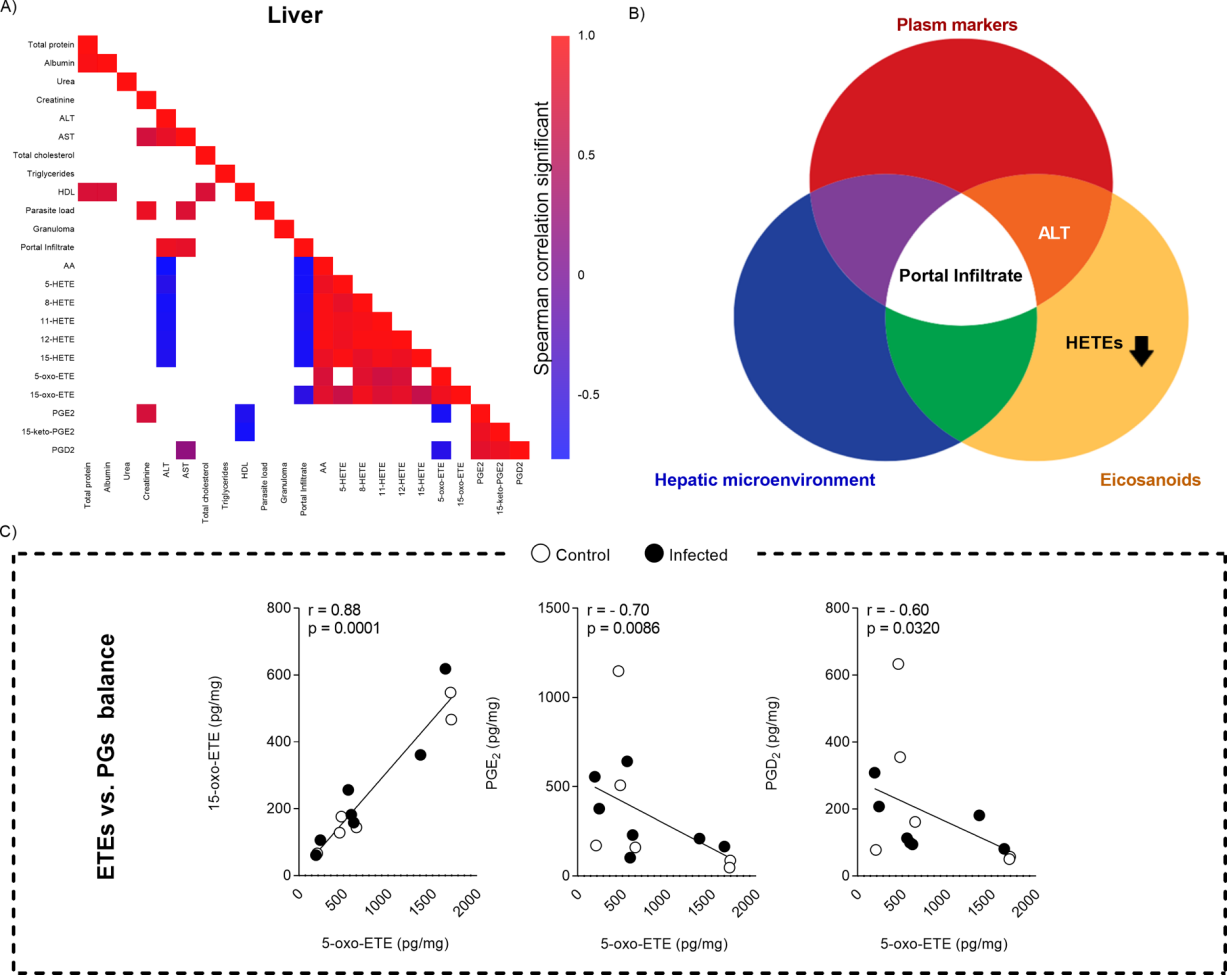
Metabolic and inflammatory changes in the liver associated with infection and parasite load. (A) Spearman correlation matrix showing the relationships between different biochemical, inflammatory and parasite load parameters in the liver (*n* = 7 animals per group). The color intensity represents the strength of the correlation, with red indicating significant positive correlations (*p* < 0.05) and blue indicating significant negative correlations (*p* < 0.05). (B) Venn diagram illustrating the intersection between various categories of biomarkers evaluated: plasma markers (red), eicosanoids (orange) and the hepatic microenvironment (blue). (C) The graphs show the relationship between the levels of 5-oxo-ETE and other lipid metabolites in the liver, comparing the control (white circles) and infected (black circles) groups. The *X*-axes represent the concentration of 5-oxo-ETE (pg/mg), while the *Y*-axes represent the concentration of the different metabolites (pg/mg). *r* = Pearson correlation coefficient; *p* = *p* value.

**Figure 7:**
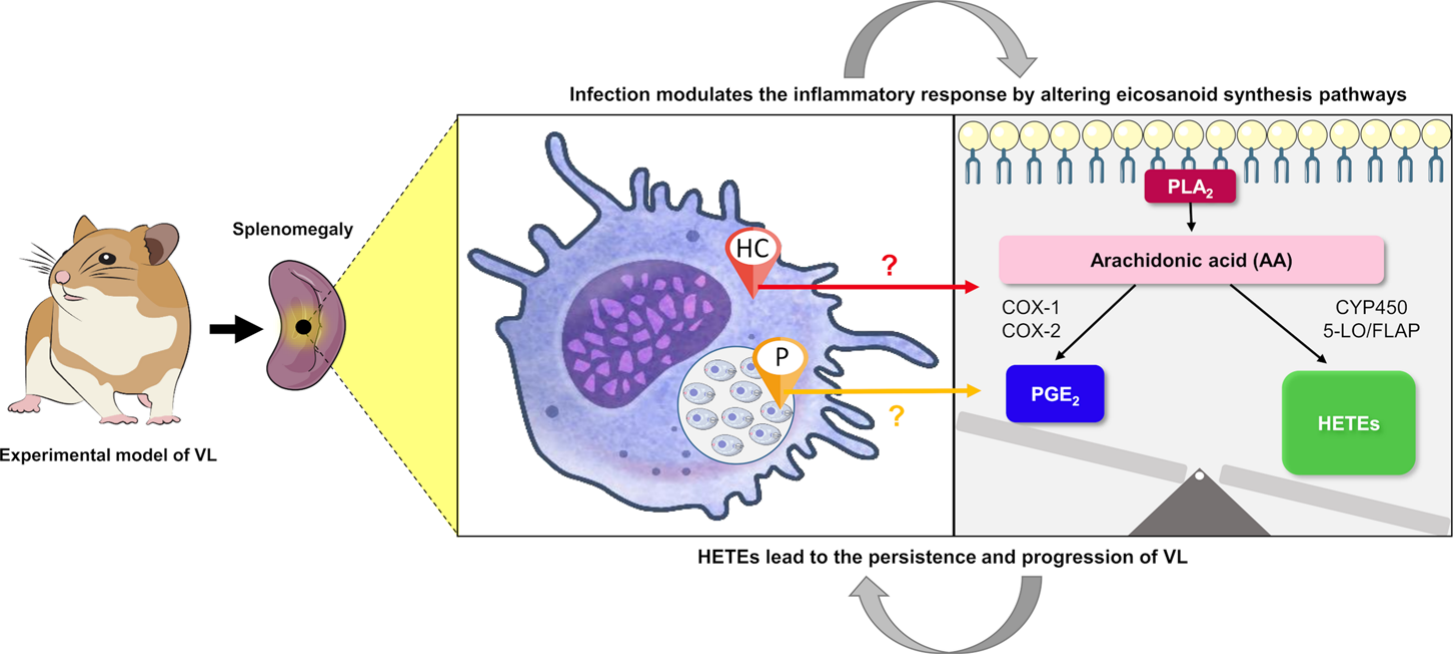
Influence of eicosanoid metabolism on the pathogenesis of VL. The experimental hamster model (Golden Syrian) was infected with *L. infantum* metacyclic promastigotes via intraperitoneal injection. The infection subsequently modulates the metabolic and inflammatory response of the organism through AA oxidation, resulting in an imbalance between the production of eicosanoids, HETEs, and PGs. This imbalance contributes to the persistence of the infection and the progression of VL. The origin of the eicosanoid synthesis observed here remains uncertain. It may originate from the host cell (red arrow), the parasite (yellow arrow), or both. HC: Host cell; P: parasite (amastigote form); PLA2: phospholipase A2; COX: cyclooxygenase; CYP450: cytochrome 450; 5-LO: 5-lipoxygenase; FLAP: lipoxygenase-activating protein; HETES: hydroxyeicosatetraenoic acids; PGs: prostaglandins. (BIOART-000188, BIOART-000312, BIOART-000288; spleen and phospholipid images was provided by Servier Medical Art is licensed under CC BY 4.0).

## Discussion

In this study, we evaluated for the first time the synthesis of eicosanoids both systemically and in tissues with the highest parasitic tropism during VL. After the infection period, infected hamsters exhibited clinical and laboratory signs, including splenomegaly, increased creatinine levels, and elevated ALT and AST transaminases. These alterations are consistent with previously reported manifestations of human and canine VL.^8,13–18^ As the structure and function of the spleen are affected during the course of many chronic diseases, this study also identified significant histological alterations in splenic tissue, including granulomas and white pulp hypoplasia. Such spleen disorganization resulting from *L. infantum* infection has already been reported in humans, dogs, and murine models and may impair the host’s ability to respond to *Leishmania* infection and other pathogens.^14,19,20^

Patterns of inflammatory response in the liver have been associated with susceptibility or resistance to VL.^21^ In this study, we identified hepatic micro alterations previously described in the literature, including the presence of granulomas and portal infiltrate.^21,22^ Granulomas help contain the infection, but *Leishmania* may use them to survive and multiply.^23^ Additionally, portal infiltrates may indicate a more effective immune response against the infection. However, symptomatic dogs with positive spleen cultures were more likely to exhibit portal inflammation compared to asymptomatic dogs. Regarding the parasite load in target tissues, it is known that a reduction in hepatic parasitism is often followed by an increase in parasite burden in the spleen,^24^ a pattern also observed in this study.

Given the importance of lipid metabolism in inflammatory processes, some studies have evaluated these molecules during VL. Alterations in fatty acids and other metabolites have been observed in various samples from mice infected with *L. donovani*, another etiological agent of VL, including the spleen, liver, brain, serum, and urine.^25^ In the serum of Golden Syrian hamsters, the most affected metabolites were glycerophospholipids, α-linolenic acid, and AA.^26^ Additionally, changes in glycerophospholipids, ceramides, and other lipids were identified in the spleen, liver, and intestine of hamsters with VL.^27^ However, analyses of tissue-derived eicosanoids in hamsters using LC–MS/MS and their relationship with other features of VL are still lacking.

Here, we observed that the HETEs were predominantly synthesized in greater amounts in the spleen of animals with VL. Moreover, little or no HETE were detected in the plasma of hamsters. These low plasma concentrations indicate the absence of a significant systemic effect, with inflammation remaining restricted to the affected tissue. This occurs because HETEs primarily act in an autocrine and paracrine manner, meaning they exert their effects locally, either on the cells that produce them or on nearby cells, without the need for systemic transport. Additionally, they are highly reactive lipid metabolites with a short half-life in plasma, which limits their systemic distribution.^28,29^ A simultaneous analysis of eicosanoid profiles in plasma and tissues could offer valuable insights for developing more precise immunomodulatory treatments. However, dynamically monitoring eicosanoid levels across different biological compartments and accurately assessing plasma mediators as indicators of the tissue environment remain significant challenges.

HETEs are metabolites of AA that are oxidized by LOX or CYP450 enzymes.^30^ Since LOX and CYP450 enzymes are widely distributed across various organs and tissues, HETEs play crucial roles in normal physiological and pathophysiological processes.^31^ HETEs modulate inflammation by recruiting leukocytes and regulating endothelial cells,^32^ but their role in chronic diseases is still unclear.

Our data also showed a strong relationship between infection, the inflammatory process, and AA metabolism, which emerges as a central element in these interactions. This metabolism regulated the immune response and disease progression and was also associated with parasite load and splenomegaly. The relationship between eicosanoid levels and disease progression has been extensively studied to clarify their role in the pathogenesis of various diseases. Studies suggest that increased levels of certain 5-HETE, 12-HETE, 15-HETE and 20-HETE may be associated with insulin resistance and inflammation related to obesity and diabetes.^31,33^ Dysregulated production of HETEs has also been linked to chronic inflammatory conditions such as rheumatoid arthritis and asthma, where they contribute to the exacerbation of inflammation and tissue damage.^34,35^ In COVID-19, increased LOX-5 and CYP enzyme activity has been linked to severe disease in hospitalized patients.^36^ Additionally, elevated levels of 5-HETE, 12-HETE, and 15-HETE have been detected in the lungs of intubated patients.^37^

Although the effects of HETEs have been investigated in various pathological conditions affecting different tissues,^38–43^ evidence of their involvement in infectious diseases remains limited, with most reports associated with bacterial infections.^44–46^ To our knowledge, this study is among the few that report the participation of HETEs in infections caused by *Leishmania*, highlighting the need for further studies to better elucidate their signaling and specific effects across different cell types and tissues.

In this study, the PGs levels in the spleen of hamsters with VL were significantly lower than those of HETEs, and no PGs were detected in their plasma. PGs, such as PGE_2_, PGD_2_, PGI_2_, PGF_2α_, are also metabolites of AA, synthesized by COX-1 and COX-2 enzymes. In particular, PGE_2_ can have both pro- and antiinflammatory functions depending on the specific cellular receptors they bind to (EP1, EP2, EP3, or EP4).^47–49^ The literature demonstrates the involvement of PGs in the pathogenicity of the leishmaniasis and of the other chronic diseases caused by trypanosomatids.^50,51^ A distinct expression pattern of genes involved in the AA cascade was observed between patients with localized cutaneous leishmaniasis (LCL) and mucocutaneous leishmaniasis (MCL). This divergence correlates with decreased levels of PGE_2_ in MCL patients.^52^ In human VL, a distinct biosignature has been identified in patients, characterized by elevated serum levels of PGF_2α_ and other mediators.^8^ This biosignature was associated with the active phase of the disease, as leishmanicidal treatment gradually reversed the relationships between these markers and other evaluated parameters.^8^ In canine VL, lower serum levels of PGE_2_ in dogs infected with *L. infantum* were linked to disease severity.^9^ Additionally, a distinct biosignature differentiating resistant and susceptible dogs correlated serum PGE_2_ levels with splenic parasite load and intensity of exposure to the sand fly.^10^ An ex vivo study showed that PGE_2_ production was stimulated by *Lutzomyia longipalpis* salivary gland sonicate (SGS) and was associated with the survival of *L. infantum* in peritoneal leukocytes, making it a critical factor driving immune evasion of parasite.^53^ Although interventions targeting the PG pathway may influence the progression of leishmaniasis, further research is needed to develop more precise and effective therapeutic approaches.

To fight infections, the immune system must balance inflammatory mediators.^54,55^ In this study, we observed an imbalance in the synthesis of HETEs and tissue PGs, indicating an altered inflammatory pattern in VL that could impact disease progression. The imbalance between PGE_2_ and LTB_4_ levels and its impact on leishmaniasis has been widely studied.^8,56–61^ Studies indicate that, in infections caused by *L. amazonensis* and *L. braziliensis*, increased PGE_2_ production is linked to the suppression of the host’s immune response, while higher LTB_4_ levels activate the inflammatory mechanisms needed to eliminate the parasite.^62–65^ Therefore, understanding how different eicosanoids are made and how they work is key to creating treatments that control inflammation, kill the parasite, and protect tissues.

Parasites of the *Leishmania* genus also have the necessary machinery to synthesize eicosanoids.^12,57,66^ While host cells use eicosanoids to regulate inflammatory responses aimed at pathogen elimination,^43^ the eicosanoids produced by *Leishmania* aim to modulate the host inflammatory response to favor their survival.^66,67^ A study by Andrade et al. (2023) demonstrates that eicosanoid production can be differentially modulated by *Leishmania* species-dependent PUFAs. Additionally, in axenic *L. infantum* cultures, stimulation with AA has been shown to enhance the production of 5-HETE, 8-HETE, 11-HETE, 12-HETE, and 15-HETE, while comparatively lower levels of PGD_2_, PGE_2_, 15-keto-PGE_2_, and PGF_2α_ were observed.^12^ *Leishmania*-produced HETEs increase the parasite load in the macrophages and they are also responsible for modulating the response of infected macrophages to an M2 profile.^67^ Therefore, the possibility that some of the tissue HETEs detected in this study originate directly from the parasites cannot be excluded. Consequently, identifying its source could offer new insights into parasite immune evasion mechanisms and pave the way for targeted therapeutic strategies.

Although this study has provided valuable insights into eicosanoid synthesis in VL, several important questions remain, particularly regarding the specific mechanisms and precise sources of HETEs in tissues. Further research is needed to explore the expression of HETEs receptors on various immune cells and to develop targeted antagonists, which would help clarify the role of HETEs in VL. Regarding their translational potential, the direct quantification of HETEs in tissues such as the spleen still faces significant barriers to clinical application, owing to both the invasive nature of sample collection and the complexity of the analyses. Nevertheless, the data presented here provide relevant evidence for the role of these eicosanoids in the pathophysiology of VL, possibly reflecting a persistent and dysregulated inflammatory environment. This finding strengthens the perspective of their future use as biomarkers of disease severity or chronicity. Furthermore, understanding these gaps may pave the way for the development of diagnostic tools capable of providing crucial information on disease progression and treatment efficacy.

## Conclusion

This study highlights the critical role of AA metabolism and eicosanoid imbalance in the pathogenesis of VL. The increased HETEs and reduced prostaglandins are linked to higher parasite load, spleen damage, and inflammation. These findings suggest that targeting eicosanoid pathways, particularly the HETEs/PGs balance, could be a promising therapeutic strategy. Understanding the host-parasite interplay in lipid signaling may pave the way for novel biomarkers and more effective interventions to modulate inflammation, control parasitism, and mitigate tissue damage in VL.

## Methods

### Cultivation of *L. infantum*

*L. infantum* promastigotes were cultured in a modified hemoflagellate cultivation medium (HO-MEM) supplemented with 10% fetal bovine serum and incubated in a BOD chamber at 26 °C, reaching the stationary phase after 7–9 days.^68^

### Hamster Infection

Golden Syrian hamsters (*M. auratus*) were used in this study. A total of 7 animals per group were used. The animals were obtained from the colony at the Gonçalo Moniz Research Center following approval by the Ethics Committee for Animal Use (CEUA), under protocol #004/2020. They were housed in the experimental facilities of the same vivarium, provided with species-specific food and water ad libitum, and maintained under controlled conditions of temperature and light/dark cycles. At 6–8 weeks of age, the male hamsters were inoculated via intraperitoneal injection into the lower right quadrant with either a saline solution or 10^7^ promastigotes/mL of *L. infantum*.

After 150 days (five months) of infection, the hamsters were anesthetized with ketamine (100 mg/kg) and xylazine (5 mg/kg)^69^ and underwent clinical evaluation. The following parameters were measured: total body weight, skin or mucous membrane lesions, and alopecia. Blood was then collected via cardiac puncture and stored in tubes without EDTA. Following blood collection, a lethal dose of ketamine (600 mg/kg) and xylazine (30 mg/kg) was administered. Necropsy was performed, including the measurement of the spleen and liver weight and dimensions. These organs were then harvested and stored for subsequent analysis.

### Biochemical Analyses

Blood samples collected via cardiac puncture were centrifuged at 12,000 rpm for 10 min to obtain serum. These samples were then sent to a veterinary hospital (HOSPMEV) for the measurement of total protein, albumin, urea, creatinine, aspartate aminotransferase (AST), alanine aminotransferase (ALT), cholesterol, triglycerides, and high-density lipoprotein (HDL). The tests were conducted using a spectrophotometric identification system on a semiautomated biochemical analyzer (Cobas Mira-Roche Diagnostic System), employing standardized commercial kits for animals from Bioclin/Biosystems or Lab-test.

### Histopathology

Fragments of spleen and liver collected during necropsy were initially fixed in 10% buffered formalin at room temperature for 48 h. Tissue samples, approximately 3–4 mm thick, were then placed in histological processing cassettes, embedded in paraffin, and sectioned into 3–4 μm slices. The sections were stained with hematoxylin and eosin. The slides were then examined by an experienced pathologist, who assessed the presence of granulomas and the proportion of white pulp in the spleen, as well as granulomas and foci of intrasinusoidal leukocytosis in the liver. The frequency of these events was determined by analyzing one slide per animal, each containing three tissue sections. Finally, the slides were digitized, and morphometric analyses were conducted using the VSViewer MetaSystems Image Viewer, version 2.0.

### Parasite Load

The parasite load in the spleen and liver of hamsters was determined by a duplex qPCR, which simultaneously detects *L. infantum* kinetoplast DNA (kDNA) and a conserved region of the mammalian housekeeping gene 18S rRNA,^70^ as briefly described below. DNA extraction from 10 mg of spleen and 25 mg of liver was performed according to the manufacturer’s instructions (DNeasyR Blood & Tissue KIT, Qiagen). Then, the total DNA extracted from the samples was quantified, using 10 ng/μL of DNA from each sample for qPCR. The oligonucleotides for detecting *L. infantum* kDNA were: LEISH-1, 5′-AACTTTTCTGGTCCCCGGGTAG-3′; LEISH-2, 5′-ACCCCCAGTTTCCCGCC-3′; and LEISH-P, 5′-FAM-AAAATGGGTGCAGAAAT-MGB/NFQ-3′.^71^ Oligonucleotides for amplification of the housekeeping sequence: 18S_F, 5′-TGCGAATGGCTCATTAAATC-3′; 18S_R, 5′-CGTCGGCATGTATTAGCTCT-3′; and 18S_P, 5′-HEX-TGGTTCCTTTGGTCGCTCGCT-BHQ1–3′.^70^ The duplex qPCR contained 5 μL of extracted DNA, Multiplex PCR Mastermix (IBMP/Fiocruz-PR, Brasil), 160 nM de 18S_F e 18S_R, 40 nM de 18S_P, 200 nM de LEISH-P and 900 nM of LEISH-1 and LEISH-2.^70^ Reactions were performed in duplicate and run on an ABI7500 Fast Real-Time PCR system (Life Technologies, EUA) configured with the following protocol: 1 × 95 °C/10 min; 45× [95 °C/15 s, 60 °C/60 s]. Data were expressed as mean cycle threshold (*C*_t_) values. Positivity for *L. infantum* DNA in the duplex qPCR reaction was determined by Ct cutoff values obtained using a receiver operating characteristic (ROC) curve analysis for each tissue, as previously described.^72^

### Lipid Extraction for Eicosanoid Detection

Plasma samples (250 μL) and 100 mg of spleen and liver were subjected to lipid extraction, which briefly involves hypotonic lysis in a 1:1 solution of deionized water and methanol at 4 °C. The samples were then stored at −80 °C and sent for eicosanoid quantification at the Center of Excellence for Lipid Quantification and Identification (CEQIL) using the LC/MS method (Nexera-TripleTOF 5600+).

### Lipid Analysis by LC–MS

Plasma, spleen, and liver samples were spiked with internal standard (IS) solution (Cayman Chemical, Ann Arbor, Michigan), then protein precipitation was performed with 1.5 mL of methanol/acetonitrile (1:1) at 4 °C and allowed to denature overnight. Subsequently, the samples were centrifuged for 10 min at 4 °C and 800×*g*. Denatured proteins were quantified by the Bradford protein assay to normalize the lipid concentration of each sample, and the resulting supernatants were diluted with Milli-Q water to decrease the organic solvent to a maximum concentration of 10–15%. For the SPE extraction protocol, the cartridge (Hypersep C18–500 mg, 3 mL, Thermo Scientific, Bellefonte, Pennsylvania) was washed with 4 mL of MeOH and equilibrated with 4 mL of H_2_O using an extraction manifold (Waters, Milford, Connecticut). After loading the diluted samples, the cartridges were washed again with 4 mL of H_2_O to remove hydrophilic impurities. The analytes were eluted with 1 mL of MeOH. The solvent was removed in vacuo (Concentrator Plus, Eppendorf, Hamburg, Germany) at room temperature and redissolved in 50 μL of MeOH/H_2_O (7:3) for LC–MS/MS analysis. Lipid extraction samples were transferred to autosampler vials and 10 μL of each sample was injected into the target LC–MS/MS system TripleTOF 5600+ (Sciex, Foster City, California), as previously described (Sorgi et al., 2018). The method used for the high-performance liquid chromatography system (HPLC) (Nexera X2, Shimadzu, Kyoto, Japan) was made using a column Ascentis Express C18 (100 × 4.6 mm and particle size of 2.7 μm) (Supelco, St. Louis, Missouri). Elution was conducted under a binary gradient system with phase A, H_2_O/ACN/acetic acid (69.98:30:0.02) at pH 5.8, and phase B, an ACN/isopropanol (70:30). Gradient elution was performed for 25 min at a flow rate of 0.6 mL min^–1^. An electrospray ionization (ESI) source in negative ion mode was used for high-resolution multiple reaction monitoring (HRMMR) scanning. Additional instrumental parameters: nebulizer gas (GS1), 50 psi; turbo gas (GS2), 50 psi; curtain gas (CUR), 25 psi; electrospray voltage (ISVF), −4.0 kV; and turbo ion spray source temperature, 550 °C. Data acquisitions were performed using AnalystTM Software (Sciex, Foster, California). Data processing proceeded through several steps, including filtering, feature detection, alignment, and normalization. Then, PeakView 2.1 software (Sciex, Foster, California) was used to identify lipid species and MultiQuant software (Sciex, Foster, California) for quantitative analysis. The final oxylipin concentration in the samples was normalized by the protein concentration.^7^

### Statistical Analysis

Statistical analysis of group differences was performed using parametric and nonparametric tests, depending on data distribution. The unpaired Student*t*test was used to compare means, while the Mann–Whitney test was applied to compare medians. Spearman’s correlation test was used for correlation analyses. Graphs and the heatmap were generated and analyzed in GraphPad Prism (version 8.0) with statistical significance set at *p* < 0.05. Spider chart representing the comparative analysis of AA and eicosanoid production in the spleen and liver of infected and noninfected animals. The data were processed and analyzed using R software.

## Supplementary Material



## Notes

Target organs of VL, polyunsaturated fatty acid (PUFA) and eicosanoids (PDF)

## References

[1] Mathison B. A., Bradley B. T. (2023). Review of the Clinical
Presentation, Pathology, Diagnosis,
and Treatment of Leishmaniasis. Lab Med..

[2] Burza S., Croft S. L., Boelaert M. (2018). Leishmaniasis. Lancet.

[3] Melby P. C., Chandrasekar B., Zhao W., Coe J. E. (2001). The Hamster
as a
Model of Human Visceral Leishmaniasis: Progressive Disease and Impaired
Generation of Nitric Oxide in the Face of a Prominent Th1-Like Cytokine
Response. J. Immunol..

[4] Miao J., Chard L. S., Wang Z., Wang Y. (2019). Syrian Hamster as an
Animal Model for the Study on Infectious Diseases. Front. Immunol..

[5] Bozza P. T., Bakker-Abreu I., Navarro-Xavier R. A., Bandeira-Melo C. (2011). Lipid Body
Function in Eicosanoid Synthesis: An Update. Prostaglandins Leukot. Essent. Fatty Acids.

[6] Melo R. C. N., Weller P. F., De Fora J., Israel B., Medical D., Avenue B. (2016). Lipid Droplets in Leukocytes:
Organelles Linked to
Inflammatory Responses. Exp. Cell Res..

[7] Sorgi C., Peti A., Petta T., Meirelles A. F. G., Fontanari C., Moraes L. A. B. d., Faccioli L. H. (2018). Comprehensive High-Resolution
Multiple-Reaction Monitoring Mass Spectrometry for Targeted Eicosanoid
Assays. Sci. Data.

[8] Araújo-Santos T., Andrade B. B., Gil-Santana L., Luz N. F., Dos Santos P. L., De Oliveira F. A., Almeida M. L., De Santana Campos R.
N., Bozza P. T., Almeida R. P., Borges V. M. (2017). Anti-Parasite Therapy
Drives Changes in Human Visceral Leishmaniasis-Associated Inflammatory
Balance. Sci. Rep..

[9] Solcà M. S., Andrade B. B., Abbehusen M. M. C., Teixeira C. R., Khouri R., Valenzuela J. G., Kamhawi S., Bozza P. T., Fraga D. B. M., Borges V. M., Veras P. S. T., Brodskyn C. I. (2016). Circulating Biomarkers
of Immune Activation, Oxidative Stress and Inflammation Characterize
Severe Canine Visceral Leishmaniasis. Sci. Rep..

[10] Solcà M. d. S., Arruda M. R., Leite B. M. M., Mota T. F., Rebouças M. F., de Jesus M. S., Amorim L. D. A. F., Borges V. M., Valenzuela J., Kamhawi S., Veras P. S. T., Fraga D. B. M., Brodskyn C. I. (2021). Immune
Response Dynamics and Lutzomyia Longipalpis Exposure Characterize
a Biosignature of Visceral Leishmaniasis Susceptibility in a Canine
Cohort. PLoS Negl. Trop. Dis..

[11] Malta-Santos H., Fukutani K. F., Sorgi C. A., Queiroz A. T. L., Nardini V., Silva J., Lago A., Carvalho L. P., Machado P. L. R., Bozza P. T., França-Costa J., Faccioli L. H., Carvalho E. M., Andrade B. B., Borges V. M. (2020). Multi-Omic
Analyses of Plasma Cytokines,
Lipidomics, and Transcriptomics Distinguish Treatment Outcomes in
Cutaneous Leishmaniasis. iScience.

[12] Andrade Y. M. F. d. S., Castro M. V. d., Tavares V. d. S., Souza R. d. S. O., Faccioli L. H., Lima J. B., Sorgi C. A., Borges V. M., Araújo-Santos T. (2023). Polyunsaturated
Fatty Acids Alter the Formation of
Lipid Droplets and Eicosanoid Production in Leishmania Promastigotes. Mem. Inst. Oswaldo Cruz.

[13] dos-Santos W. L. C., Pagliari C., Santos L. G., Almeida V. A., e Silva T. L. V., Coutinho J. de J., Souza T., Duarte M. I. S., de Freitas L. A. R., Costa C. H. N. (2014). A Case of Conventional
Treatment Failure in Visceral Leishmaniasis: Leukocyte Distribution
and Cytokine Expression in Splenic Compartments. BMC Infect. Dis..

[14] Lima I. S., Silva J. S., Almeida V. A., Junior F. G. L., Souza P. A. N., Larangeira D. F., Moura-Neto J. P., Fraga D. B. M., De Freitas L. A. R., Dos-Santos W. L. C. (2014). Severe
Clinical Presentation of Visceral Leishmaniasis in Naturally Infected
Dogs with Disruption of the Splenic White Pulp. PLoS One.

[15] Oliveira M. J. C., Silva G. B., Abreu K. L. S., Rocha N. A., Garcia A. V. V., Franco L. F. L. G., Mota R. M. S., Libório A. B., Daher E. F. (2010). Risk Factors for
Acute Kidney Injury in Visceral Leishmaniasis
(Kala-Azar). Am. J. Trop. Med. Hyg..

[16] Dores M., Das N., Vitoriano-Souza J., Roatt B. M., De Abreu
Vieira P. M., Coura-Vital W., De Oliveira Cardoso J. M., Rezende M. T., Ker H. G., Giunchetti R. C., Carneiro C. M., Reis A. B. (2016). Clinical, Hematological and Biochemical
Alterations in Hamster (Mesocricetus Auratus) Experimentally Infected
with Leishmania Infantum through Different Routes of Inoculation. Parasites Vectors.

[17] Jiménez-Antón M. D., Grau M., Olías-Molero A.
I., Alunda J. M. (2019). Syrian
Hamster as an Advanced Experimental Model for Visceral Leishmaniasis. Methods Mol. Biol..

[18] Resende L. A., Aguiar-Soares R. D. d. O., Moreira N. d. D., Ferreira S. d. A., Lanna M. F., Cardoso J. M. d. O., Mathias F. A. S., Coura-Vital W., Mariano R. M. d. S., Leite J. C., Silveira P., de Carvalho T. F., Santos R. L., Silveira-Lemos D. da, Martins-Filho O. A., Dutra W. O., Reis A. B., Giunchetti R. C. (2020). In Vitro
Infectivity of Strains Isolated From Dogs Naturally Infected With
Leishmania Infantum Present a Distinct Pathogenic Profile in Hamsters. Front. Med..

[19] Hermida M. d. E. R., de Melo C. V. B., Lima I. D. S., Oliveira G. G. de S., Dos-Santos W. L. C. (2018). Histological Disorganization of Spleen
Compartments and Severe Visceral Leishmaniasis. Front. Cell. Infect. Microbiol..

[20] de
Melo C. V. B., Hermida M. D. E. R., Mesquita B. R., Fontes J. L. M., Koning J. J., Solcà M. da S., Benevides B. B., Mota G. B. S., Freitas L. A. R., Mebius R. E., Dos-Santos W. L. C. (2020). Phenotypical
Characterization of Spleen Remodeling in Murine Experimental Visceral
Leishmaniasis. Front. Immunol..

[21] Hag I. A. e., Hashim F. A., Toum I. A. El, Homeida M. (1994). Liver Morphology and
Function in Visceral Leishmaniasis (Kala-Azar). J. Hepatol..

[22] Lima I. S., Solcá M. S., Tafuri W. L., Dos-Santos W. L. C., De Freitas L. A. R. (2019). Assessment
of Histological Liver
Alterations in Dogs Naturally Infected with Leishmania Infantum. Parasites and Vectors.

[23] Murray H. W. (2001). Tissue
Granuloma Structure-Function in Experimental Visceral Leishmaniasis. Int. J. Exp. Pathol..

[24] Engwerda C. R., Kaye P. M. (2000). Organ-Specific Immune
Responses Associated with Infectious
Disease. Immunol. Today.

[25] Das S., Saha T., Shaha C. (2021). Tissue/Biofluid
Specific Molecular
Cartography of Leishmania Donovani Infected BALB/c Mice: Deciphering
Systemic Reprogramming. Front. Cell. Infect.
Microbiol..

[26] Qin H., Zhang J., Dong K., Chen D., Yuan D., Chen J. (2022). Metabolic
Characterization and Biomarkers Screening for Visceral
Leishmaniasis in Golden Hamsters. Acta Trop..

[27] Lesani M., Gosmanov C., Paun A., Lewis M. D., McCall L. I. (2022). Impact
of Visceral Leishmaniasis on Local Organ Metabolism in Hamsters. Metabolites.

[28] Biringer R. G. (2020). The Enzymology
of Human Eicosanoid Pathways: The Lipoxygenase Branches. Mol. Biol. Rep..

[29] Chhonker Y. S., Bala V., Murry D. J. (2018). Quantification of
Eicosanoids and
Their Metabolites in Biological Matrices: A Review. Bioanalysis.

[30] Powell W. S. (2015). Biosynthesis
Biological Effects, and Receptors of Hydroxyeicosatetraenoic Acids
(HETEs) and Oxoeicosatetraenoic Acids (Oxo-ETEs) Derived from Arachidonic
Acid. Biochim. Biophys. Acta.

[31] Dong L., Wang H., Chen K., Li Y. (2022). Roles of Hydroxyeicosatetraenoic
Acids in Diabetes (HETEs and Diabetes). Biomed.
Pharmacother..

[32] Stables M. J., Gilroy D. W. (2011). Old and New Generation Lipid Mediators
in Acute Inflammation
and Resolution. Prog. Lipid Res..

[33] Chen S., Qian Y., Lin Q., Chen Z., Xiang Z., Cui L., Sun J., Qin X., Xu Y., Lu L., Zou H. (2022). Increased Serum 12-Hydroxyeicosatetraenoic
Acid Levels Are Correlated
with an Increased Risk of Diabetic Retinopathy in Both Children and
Adults with Diabetes. Acta Diabetol..

[34] Bryda J., Wątroba S. (2018). The Proinflammatory
Role of Lipoxygenases in Rheumatoid
Arthritis. J. Pre-Clin. Clin. Res..

[35] Kytikova O. Y., Kovalenko I. S., Novgorodtseva T. P., Denisenko Y. K. (2024). The Role
of Hydroxyeicosatetraenoic Acids in the Regulation of Inflammation
in Bronchial Asthma. Dokl. Biochem. Biophys..

[36] Schwarz B., Sharma L., Roberts L., Peng X., Bermejo S., Leighton I., Casanovas-Massana A., Minasyan M., Farhadian S., Ko A. I., Dela
Cruz C. S., Bosio C. M., Haven N., Haven N. (2021). Cutting Edge:
Severe SARS-CoV-2 Infection in Humans Is Defined by
a Shift in the Serum Lipidome, Resulting in Dysregulation of Eicosanoid
Immune Mediators. J. Immunol..

[37] Archambault A. S., Zaid Y., Rakotoarivelo V., Turcotte C., Doré É., Dubuc I., Martin C., Flamand O., Amar Y., Cheikh A., Fares H., El Hassani A., Tijani Y., Côté A., Laviolette M., Boilard É., Flamand L., Flamand N. (2021). High Levels
of Eicosanoids
and Docosanoids in the Lungs of Intubated COVID-19 Patients. FASEB J..

[38] Ni K. D., Liu J. Y. (2021). The Functions
of Cytochrome P450 ω-Hydroxylases
and the Associated Eicosanoids in Inflammation-Related Diseases. Front. Pharmacol.

[39] Huang H., Al-Shabrawey M., Wang M. H. (2016). Cyclooxygenase-
and Cytochrome P450-Derived
Eicosanoids in Stroke. Prostaglandins Other
Lipid Mediators.

[40] Afshinnia F., Zeng L., Byun J., Wernisch S., Deo R., Chen J., Hamm L., Miller E. R., Rhee E. P., Fischer M. J., Sharma K., Feldman H. I., Michailidis G., Pennathur S. (2020). Elevated Lipoxygenase and Cytochrome P450 Products
Predict Progression of Chronic Kidney Disease. Nephrol. Dial. Transplant..

[41] Perepechaeva M. L., Grishanova A. Y. (2024). The Role of Arachidonic Acid Metabolizing Cytochromes
P450 in the Control of Cardiovascular Functions. Biochem. Suppl. Ser. B Biomed. Chem..

[42] Atone J., Wagner K., Hashimoto K., Prostaglandins B. D. H. (2020). Cytochrome
P450 Derived Epoxidized Fatty Acids as a Therapeutic Tool against
Neuroinflammatory Diseases. Prostag. Other Lipid
Mediat..

[43] Dennis E. A., Norris P. C. (2015). Eicosanoid Storm
in Infection and Inflammation. Nat. Rev. Immunol..

[44] Clark S. R., Guy C. J., Scurr M. J., Taylor P. R., Kift-Morgan A. P., Hammond V. J., Thomas C. P., Coles B., Roberts G. W., Eberl M., Jones S. A., Topley N., Kotecha S., O’Donnell V. B. (2011). Esterified
Eicosanoids Are Acutely Generated by 5-Lipoxygenase
in Primary Human Neutrophils and in Human and Murine Infection. Blood.

[45] Sheppe A. E. F., Edelmann M. J. (2021). Roles of Eicosanoids in Regulating
Inflammation and
Neutrophil Migration as an Innate Host Response to Bacterial Infections. Infect. Immun..

[46] Schultz D., Surabhi S., Stelling N., Rothe M., Methling K., Hammerschmidt S., Siemens N., Lalk M. (2020). 16HBE Cell Lipid Mediator
Responses to Mono and Co-Infections with Respiratory Pathogens. Metabolites.

[47] Fraga-Silva T. F. d.
C., Maruyama S. R., Sorgi C. A., Russo E. M. d. S., Fernandes A. P. M., de Barros Cardoso C.
R., Faccioli L. H., Dias-Baruffi M., Bonato V. L. D. (2021). COVID-19: Integrating the Complexity
of Systemic and Pulmonary Immunopathology to Identify Biomarkers for
Different Outcomes. Front. Immunol..

[48] Ricciotti E., FitzGerald G. A. (2011). Prostaglandins
and Inflammation. Arter. Thromb Vasc Boil.

[49] Pereira P. A. T. (2018). Prostaglandins D 2 and
E 2 Have Opposite Effects on
Alveolar Macrophages Infected with Histoplasma Capsulatum. J. Lipid Res..

[50] Brodskyn C. I., Kamhawi S. (2018). Biomarkers for Zoonotic Visceral Leishmaniasis in Latin
America. Front. Cell. Infect. Microbiol..

[51] López-Muñoz R. A., Molina-Berríos A., Campos-Estrada C., Abarca-Sanhueza P., Urrutia-Llancaqueo L., Peña-Espinoza M., Maya J. D. (2018). Inflammatory and Pro-Resolving Lipids
in Trypanosomatid
Infections: A Key to Understanding Parasite Control. Front. Microbiol..

[52] França-Costa J., Andrade B. B., Khouri R., Van Weyenbergh J., Malta-Santos H., Da Silva Santos C., Brodyskn C. I., Costa J. M., Barral A., Bozza P. T., Boaventura V., Borges V. M. (2016). Differential Expression of the Eicosanoid Pathway in
Patients with Localized or Mucosal Cutaneous Leishmaniasis. J. Infect. Dis..

[53] Araújo-Santos T., Prates D. B., França-Costa J., Luz N. F., Andrade B. B., Miranda J. C., Brodskyn C. I., Barral A., Bozza P. T., Borges V. M. (2014). Prostaglandin E2/Leukotriene B4 Balance Induced by
Lutzomyia Longipalpis Saliva Favors Leishmania Infantum Infection. Parasites and Vectors.

[54] Chen L., Deng H., Cui H., Fang J., Zuo Z., Deng J., Li Y., Wang X., Zhao L. (2018). Inflammatory
Responses and Inflammation-Associated Diseases in Organs. Oncotarget.

[55] Medzhitov R. (2008). Origin and
Physiological Roles of Inflammation. Nature.

[56] Malta-Santos H., Fukutani K. F., Sorgi C. A., Queiroz A. T. L., Nardini V., Silva J., Lago A., Carvalho L. P., Machado P. L. R., Bozza P. T., França-Costa J., Faccioli L. H., Carvalho E. M., Andrade B. B., Borges V. M. (2020). Multi-Omic
Analyses of Plasma Cytokines,
Lipidomics, and Transcriptomics Distinguish Treatment Outcomes in
Cutaneous Leishmaniasis. iScience.

[57] Araújo-Santos T., Rodríguez N. E., Moura-Pontes S., Dixt U. G., Abánades D. R., Bozza P. T., Wilson M. E., Borges V. M. (2014). Role of Prostaglandin
F2α Production in Lipid Bodies from Leishmania Infantum Chagasi:
Insights on Virulence. J. Infect. Dis..

[58] Bonyek-Silva I., Nunes S., Santos R. L., Lima F. R., Lago A., Silva J., Carvalho L. P., Arruda S. M., Serezani H. C., Carvalho E. M., Brodskyn C. I., Tavares N. M. (2020). Unbalanced Production
of LTB4/PGE2 Driven by Diabetes Increases Susceptibility to Cutaneous
Leishmaniasis. Emerg. Microbes Infect..

[59] Chaves M. M., Canetti C., Coutinho-Silva R. (2016). Crosstalk
between Purinergic Receptors
and Lipid Mediators in Leishmaniasis. Parasites
and Vectors.

[60] França-Costa J., Andrade B. B., Khouri R., Van Weyenbergh J., Malta-Santos H., Da Silva Santos C., Brodyskn C. I., Costa J. M., Barral A., Bozza P. T., Boaventura V., Borges V. M. (2016). Differential Expression of the Eicosanoid
Pathway in
Patients with Localized or Mucosal Cutaneous Leishmaniasis. J. Infect. Dis..

[61] Tavares N. M., Araújo-Santos T., Afonso L., Nogueira P. M., Lopes U. G., Soares R. P., Bozza P. T., Bandeira-Melo C., Borges V. M., Brodskyn C. (2014). Understanding the Mechanisms Controlling
Leishmania Amazonensis Infection in Vitro: The Role of LTB4derived
from Human Neutrophils. J. Infect. Dis..

[62] Morato C. I., da Silva I. A., Borges A. F., Dorta M. L., Oliveira M. A. P., Jancar S., Serezani C. H., Ribeiro-Dias F. (2014). Essential
Role of Leukotriene B4 on Leishmania (Viannia) Braziliensis Killing
by Human Macrophages. Microb. Infect..

[63] Tavares N., Afonso L., Suarez M., Ampuero M., Prates D. B., Araújo-Santos T., Barral-Netto M., DosReis G. A., Borges V. M., Brodskyn C. (2016). Degranulating
Neutrophils Promote Leukotriene B4 Production
by Infected Macrophages To Kill Leishmania Amazonensis Parasites. J. Immunol..

[64] Chaves M. M., Marques-da-Silva C., Monteiro A. P. T., Canetti C., Coutinho-Silva R. (2014). Leukotriene
B4Modulates P2 × 7 Receptor–Mediated Leishmania Amazonensis
Elimination in Murine Macrophages. J. Immunol..

[65] Serezani C. H., Perrela J. H., Russo M., Peters-Golden M., Jancar S. (2006). Leukotrienes Are Essential for the
Control of Leishmania
Amazonensis Infection and Contribute to Strain Variation in Susceptibility. J. Immunol..

[66] Tavares V. D. S., De Castro M. V., Souza R. D. S. O., Gonçalves I. K. A., Lima J. B., Borges V. D. M., Araújo-Santos T. (2021). Lipid Droplets
from Protozoan Parasites: Survival and Pathogenicity. Mem. Inst. Oswaldo Cruz.

[67] Paloque L., Perez-Berezo T., Abot A., Dalloux-Chioccioli J., Bourgeade-Delmas S., Le Faouder P., Pujo J., Teste M. A., François J. M., Schebb N. H., Mainka M., Rolland C., Blanpied C., Dietrich G., Bertrand-Michel J., Deraison C., Valentin A., Cenac N. (2019). Polyunsaturated Fatty
Acid Metabolites: Biosynthesis in Leishmania and Role in Parasite/Host
Interaction. J. Lipid Res..

[68] Yao C., Chen Y., Sudan B., Donelson J. E., Wilson M. E. (2008). Leishmania
Chagasi: Homogenous Metacyclic Promastigotes Isolated by Buoyant Density
Are Highly Virulent in a Mouse Model. Exp. Parasitol..

[69] Flecknell P., Lofgren J. L. S., Dyson M. C., Marini R. R., Michael
Swindle M., Wilson R. P. (2015). Preanesthesia, Anesthesia, Analgesia,
and Euthanasia. Lab. Anim. Med..

[70] Rampazzo R., Solcà M. d. S., Santos L. C. S., Pereira L. d. N., Guedes J. C. O., Veras P. S. T., Fraga D. B. M., Krieger M. A., Costa A. D. T., Costa A. D. T. (2017). A Ready-to-Use Duplex QPCR to Detect
Leishmania Infantum DNA in Naturally Infected Dogs. Vet. Parasitol..

[71] Francino O., Altet L., Sánchez-Robert E., Rodriguez A., Solano-Gallego L., Alberola J., Ferrer L., Sánchez A., Roura X. (2006). Advantages of Real-Time PCR Assay
for Diagnosis and Monitoring of
Canine Leishmaniosis. Vet. Parasitol..

[72] Solcà M. d. S., Bastos L. A., Guedes C. E. S., Bordoni M., Borja L. S., Larangeira D. F., Da Silva Estrela Tuy P. G., Amorim L. D. A. F., Nascimento E. G., de Sá Oliveira G. G., Dos-Santos W. L. C., Fraga D. B. M., Veras P. S. T. (2014). Evaluating the
Accuracy of Molecular Diagnostic Testing for Canine Visceral Leishmaniasis
Using Latent Class Analysis. PLoS One.

