# Supplementary material for: Arachidonic Acid Metabolism and HETEs-PGs Imbalance in L. infantum Infection: Implications for Visceral Leishmaniasis Progression
Source: ACS Omega. 2025 Sep 12;10(38):43767–78. doi: 10.1021/acsomega.5c04179 (PMC12489632; doi:10.1021/acsomega.5c04179)
Supplement: Supplementary file 1 [file ao5c04179_si_001.pdf]

Support information

Arachidonic acid metabolism and HETEs-PGs imbalance in *L. infantum* infection: implications for Visceral Leishmaniasis progression

Yasmin Monara Ferreira de Sousa Andrade[a*]; Astrid Madeleine Calero Goicochea[b,c]; Flávio Henrique Jesus-Santos[d]; Jonathan Luís Magalhães Fontes[b,c]; Bianca Ramos Mesquita[b,c]; Caroline Vilas Boas de Melo[b,c]; Nicole Hlavac[d]; Icaro Bonyek-Silva[b]; Manuela da Silva Solcà[b,c]; Deborah Bittencourt Mothé Fraga[b,c]; Adriana Ferreira Lopes Vilela[e]; Carlos Arterio Sorgi[e]; Washington Luis Conrado dos Santos[b,c]; Théo Araújo-Santos[a*]; Valeria M. Borges[b,c*]

[a] *Federal University of Western Bahia, Barreiras, 47808-021, Brazil*

[b] *Gonçalo Moniz Institute, Oswaldo Cruz Foundation, Salvador, 40296-710, Brazil*

[c] *Federal University of Bahia, Salvador, 40170-110, Brazil*

[d] *Federal University of Rio Grande do Sul, 90040-060, Brazil*

[e] *University of São Paulo, Ribeirão Preto, 14040-903, Brazil*

*Email: valeriaborges.br@gmail.com;

theo.santos@ufob.edu.br;

yasmin.andrade@ufob.edu.br

SUPPLEMENTARY MATERIAL

Lipids	Internal Standards	Mass (*m/z*)		Lipid concentrations					
				Spleen (pg/mg)		Liver (pg/mg)		Plasma (ng/mL)	
		Precursor ion (*m/z*)	Fragment ion (*m/z*)	Control	Infected	Control	Infected	Control	Infected
AA	5-HETE-d8	319	175.1461 - 175.1901	9567 ± 4361	42199 ± 48346**	12329667 ± 7298178	9193286 ± 4410743	331.6 ± 265.1	215.2 ± 136.9
5-HETE	5-HETE-d8	319	115.0315 - 115.0355	523.7 ± 487.8	1312 ± 521.7*	336785 ± 164585	284129 ± 147260	1.109 ± 1.160	0.1459 ± 0.1654
8-HETE	12-HETE-d8	319	155.0720 - 155.0780	100.3 ± 87.80	498.5 ± 292.0**	76522 ± 51942	52051 ± 32922	ND	ND
11-HETE	12-HETE-d8	319	167.1096 - 167.1146	224.5 ± 185.6	869.6 ± 427.4**	98840 ± 50742	71140 ± 35379	ND	ND
12-HETE	12-HETE-d8	327	184.1419 - 184.1459	190.5 ± 145.3	752.5 ± 372.6**	92807 ± 56362	72030 ± 29884	0.4367 ±0.4469	0.2020± 0.2794
15-HETE	15-HETE-d8	319	175.1461 - 175.1901	668.2 ± 685.7	1638 ± 704.7*	263198 ± 110779	217800 ± 132727	ND	ND
LTB4	12-epi-LTB4-d4	335	129.0535 - 129.0585	10019 ± 18152	197732 ± 152368*	ND	ND	ND	ND
5-oxo-ETE	5-oxo-ETE-d7	317	203.1843 - 203.1883	231.8 ± 230.9	324.7 ± 106.5	866.6 ± 666.5	743.3 ± 553	ND	ND
15-oxo-ETE	5-oxo-ETE-d7	317	113.0989 - 113.1029	128.4 ± 101.8	246.5 ± 97.95	254.9 ± 200.4	249.1 ± 190.4	ND	ND
PGE2	PGE2-d4	351	189.1301 - 189.1341	1110 ± 516.2	393.1 ± 214.4**	353.8 ± 422	326.3 ± 205.8	ND	ND
15-keto-PGE2	PGE2-d4	349	287.2055 - 287.2095	416.9 ± 194.5	124.1 ± 74.19**	574.7 ± 520.3	504.2 ± 209.6	ND	ND
PGF2a	PGF2a-d4	353	309.2021 - 309.2061	586 ± 310	372.4 ± 87.49	ND	ND	ND	ND
PGB2	PGE2-d4	333	175.1136 - 175.1176	368.9 ± 398.7	144.3 ± 91.93	ND	ND	ND	ND
PGD2	PGD2-d4	351	189.1300 - 189.1340	737.8 ± 355.3	248.8 ± 162.9**	222.7 ± 231.6	155.1 ± 82.76	ND	ND

Supplementary Table 1. Quantitation of eicosanoids in spleen, liver and plasm of Golden Syrian Hamster with VL by LC-MS/MS. As the data follow a Gaussian distribution, values are expressed as mean ± standard deviation (SD) and were analyzed using T-tests for eicosanoid quantification in infected and uninfected animals. P-values are represented as follows: *$p < 0.05$; **$p < 0.01$; ***$p < 0.001$. ND: not detected.
